# An invasive plant experiences greater benefits of root morphology from enhancing nutrient competition associated with arbuscular mycorrhizae in karst soil than a native plant

**DOI:** 10.1371/journal.pone.0234410

**Published:** 2020-06-09

**Authors:** Tingting Xia, Yongjian Wang, Yuejun He, Changbang Wu, Kaiping Shen, Qiyu Tan, Liling Kang, Yun Guo, Bangli Wu, Xu Han

**Affiliations:** 1 Forestry College, Research Center of Forest Ecology, Institue for Forest Resources & Environment of Guizhou, Guizhou University, Guiyang, China; 2 College of Horticulture and Forestry Sciences / Hubei Engineering Technology Research Center for Forestry Information, Huazhong Agricultural University, Wuhan, China; Shandong University, CHINA

## Abstract

The *Eupatorium adenophorum* have widespread invaded the karst ecosystem of southwest China and threatened the regional native community stability. Arbuscular mycorrhizae (AM) plays an important role in promoting growth for host plants via root external mycelia. However, whether AM regulates plant root traits underlying competition between invasive and native species via mycorrhizal networks in karst habitats, remains unclear. An experiment was conducted in a microcosm composed of two planting compartments flanking a competition compartment. The invasive *E*. *adenophorum* and native *Artemisia annua* were each placed in one of the two planting compartments with or without *Glomus etunicatum* fungus. The nutrient access treatments included the competitive utilization (Cu), single utilization (Su) and non-utilization (Nu) by using different nylon meshes allowed or prevented mycelium passing to acquire nutrients from the competition compartment. Root traits and nutrients of the two species were analyzed. The results showed that AM fungi had differential effects on root traits and nutrients of *E*. *adenophorum* and *A*. *annua* seedlings, which increased dry weight, length, surface area, volume, tips and branching points in roots, specific root length and volume, root nitrogen (N) and phosphorus (P) contents under Cu, Su and Nu treatments. AM fungus was also associated with decreases in the average diameter for both species. Under the Cu treatment, *E*. *adenophorum* had significantly greater length, surface area, volume, tips and branching points of roots, specific root traits, and root N and P than *A*. *annua*. AM fungi changed root phenotypes and nutrient uptake for both invasive and native plant species via interconnected mycorrhizal networks. Overall, our results suggest that through mycorrhizal networks, the invasive plant experiences greater benefits than the native plant in the nutrient competition, which fosters root morphological developments in karst soil.

## Introduction

Karst landforms develop from carbonate rock and are widely distributed in southwest China [1]. This habitat is characterized by fragile ecosystems, exposed rocks, severe soil loss and nutrient deficiencies [2, 3], and susceptibility to invasive plants. In recent years, some invasive plants such as *Eupatorium adenophorum* have successfully invaded the karst habitat in southwest China and have spread continuously [4], seriously threatening native species diversity and ecological stability. However, it is not clear how alien plants successfully invade the fragile karst habitat at present. One possible factor is that when alien plants invade a new habitat, they escape their specific soil pathogenic microorganisms from their origin, and ultimately gain a competitive advantage [5]. Harner et al. (2010) [6] argued that rhizosphere symbiotic microorganisms can help invasive plants obtain nutrition and can thereby promote the growth of invasive plants. Callaway et al. (2004a; 2004b) [7, 8] also believed that soil microorganisms are the essential factor affecting the successful invasion of alien plants, the interaction of invasive plants with soil microorganisms and its feedback plays an important role in competition leading to the replacement of native plants.

Arbuscular mycorrhizal (AM) fungi are functional microorganisms that can form mutually beneficial symbiotic relationships with more than 80% of land plants [9]. An arbuscular mycorrhizal network is formed by AM fungal mycelia, which are widely distributed in soil ecosystems and link two or more plant root systems [10]. Mycorrhizal networks can promote plant absorption of mineral nutrients in the soil and can change competition relationships among plant species [11, 12]. AM fungi have a positive feedback effect on the growth of *E*. *adenophorum* and can enhance the competitive effect of *E*. *adenophorum* on native plants [13]. Awaydul et al. (2019) [14] discovered that a mycorrhizal network preferentially transfers soil N and P to the invasive plant when invasive *Solidago canadensis* and native *Kummerowa striata* are interconnected with an arbuscular mycorrhizal network. When resources are scarce, there will be competition for aboveground light and competition for belowground water and mineral nutrients among plant species [15, 16]. However, at present, the research on invasion and AM fungi mainly focus on the effects of invasive plants on the diversity of AM fungi in invaded habitats and the effect of its feedback on aboveground competition between invasive and native plants. There is less research on the competition between belowground roots of invasive and native plant species. In the early stage of community succession, due to sufficient light resources, plants need to absorb more nutrients from the soil to promote growth, and thus the root systems of adjacent species have a fierce competition for soil water and fertilizer resources [17]. The nutrient absorption for competitiveness is proportional to the size of the plant itself in belowground competition [18, 19]. Meanwhile, the neighboring competition under limited resources is more reliable [20]. Thus the belowground competition of plants plays a crucial role in plant growth and productivity [21].

Roots can enhance a plant’s competitive advantage by adjusting its morphology when two adjacent plants compete for limited soil resources [22]. Additionally, through changes in root morphology, roots can improve the plant’s ability to absorb and utilize nutrients in the soil, which can be particularly beneficial in nutrient-deficient karst areas such as Southwest China [23]. Research has shown that plant roots in karst habitats have many branches and grow horizontally, thereby ensuring strong competitiveness for water and nutrients [24]. Xu et al. (2019) [25] believed that Chinese fir seedlings could enhance its specific root length to improve their P acquisition, when in competition with adjacent plants for a limited supply of phosphorus. Besides, root morphology and nutrients were significantly affected by soil microorganisms such as AM fungi [26]. Researches have shown that AM fungi can promote growth and change the traits of plant roots, thereby helping plants absorb more mineral elements from the soil [27, 28]. For instance, AM fungi can significantly enhance the total root length and volume of tea plants [29], and can markedly improve the P acquisition of the leguminous herb roots and invasive species [30, 31]. Yang et al. (2014) [32] believed that AM fungi directly affect the utilization efficiency of nutrients of competing for the root system, and cause different plants to have asymmetric competition trends. Also, the mycorrhizal networks can change the nutritional and phenotypic traits of plants by amplifying the nutritional competition among plant species [33]. Therefore, AM fungi can affect species competition by changing plant root morphology and nutrients. However, it is unclear how do invasive plants compete with native plants in changing root traits by nutrient uptakes through mycorrhizal networks in karst habitats. We present and test the following hypotheses: (1) AM fungi can promote roots growth and nutrient uptakes of invasive and native species in karst soil; (2) the mycorrhizal networks can enhance the competitive advantage of invasive species over native species in nutrient acquisition fostering root morphological developments in karst soil.

## Materials and methods

### The experimental growth microcosm

**Fig 1:**
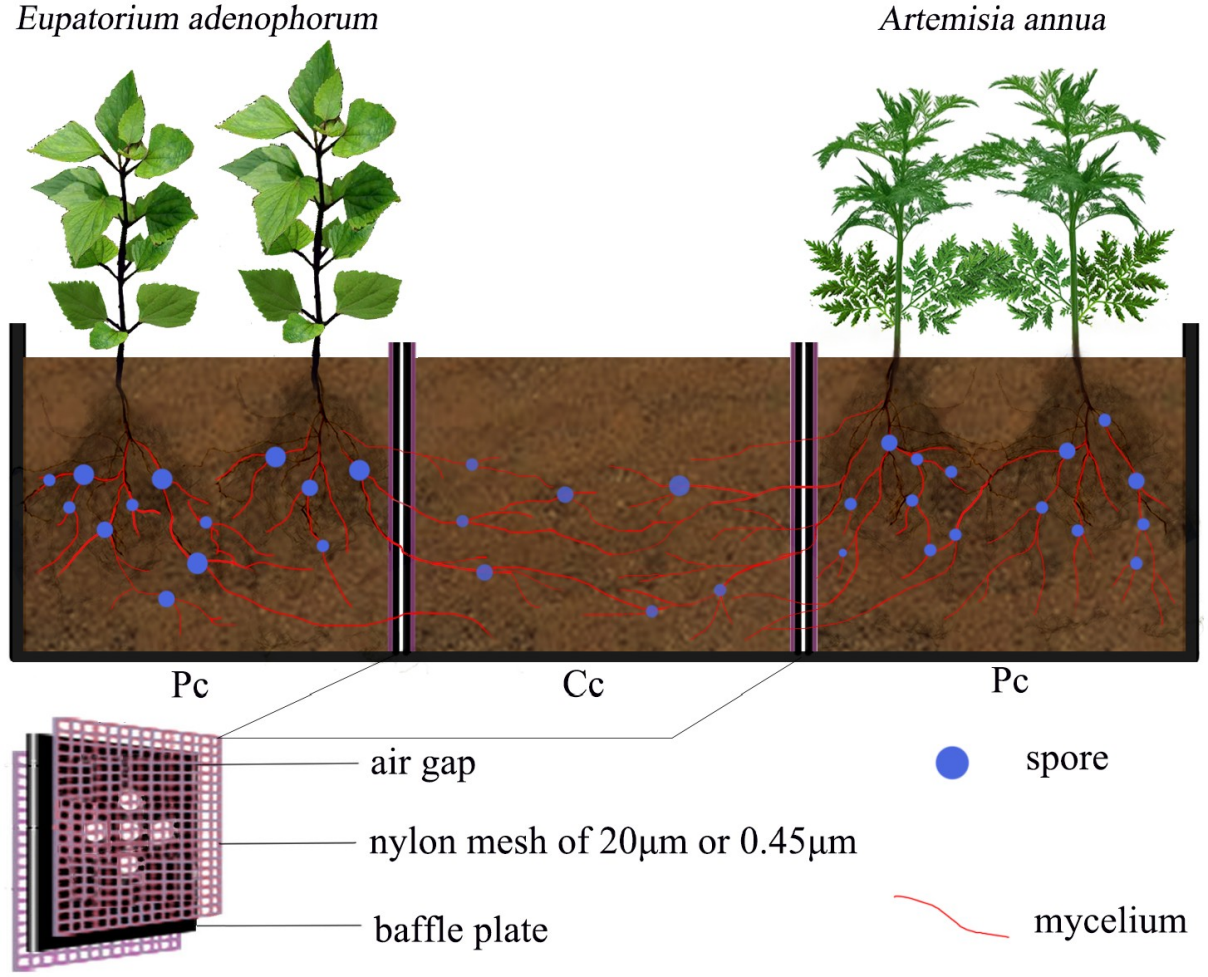
The experimental growth microcosmic device. The experimental device consists of three compartments, two planting compartments (one for the native *E*. *adenophorum* and one for the invasive *A*. *annua*) on opposite sides flanking one competition compartment in the center. The size of each of the three compartments was about 10 cm × 10 cm × 10 cm (length × width × height). Five circular holes were drilled on a baffle plate separating the planting compartments and the competition compartment. 20μm or 0.45μm nylon meshes were attached to both sides of the baffle plate to form an air gap to prevent the flow of nutrients among compartments. The 20μm nylon mesh allows mycelium to pass through, but the plant roots cannot; the 0.45μm nylon mesh does not allow either mycelium nor plant roots to pass through. More detailed description in the text. Pc = planting compartment; Cc = competition compartment.

A microcosm experiment was set up using devices with three compartments (Fig 1) in the greenhouse of Forestry College of Guizhou University, Guiyang, China (106°22′ E, 29°49′ N, 1120 m above the sea level). The experimental material was 2 mm thick polypropylene plastic. The device was composed of two planting compartments (Pc) on opposite sides flanking a competition compartment (Cc) in the center. The size of each of the three compartments was about 10 cm × 10 cm × 10 cm (length × width × height). Five circular holes with a diameter of 5 mm were drilled on a baffle plate separating the planting compartments and the competition compartment. 20μm or 0.45μm nylon meshes were attached to both sides of the baffle plate to form an air gap to prevent nutrient flow exchanges among compartments. Additionally, the 20μm Glomusnylon mesh allows mycelium to pass through, but the plant roots cannot, and the 0.45μm nylon mesh does not allow either mycelium nor plant roots to pass through [34].

This experiment had both mycorrhizal fungus treatments and nutrient access treatments. The mycorrhizal fungus treatments (M^+^) included inoculation with 50g *Glomus etunicatum* inoculum (The *Glomus etunicatum* has a new name, *Claroideoglomus etunicatum* [35], purchased from the Institute of Nutritional Resources, Beijing Academy of Agricultural and Forestry Sciences, BGGAM0046), and the control without *Glomus etunicatum* (M^-^), containing probable 500 spores as well as hyphae and colonized root pieces. The nutrient access treatments for the competition compartment interconnecting two planting compartments by mycorrhizal networks, which used 20um nylon mesh and 0.45um nylon mesh in different ways. As follows: (1) the competitive utilization nutrient treatment (Cu) was to use the 20μm double-nylon mesh on the baffle plate of the central competition compartment and the planting compartments on both sides, allowing the AM mycelia in planting compartments of *E*. *adenophorum* and *A*. *annua* commonly entering into the competition compartment, and form an interconnected mycorrhizal networks among all three compartments; (2) the single utilization nutrient treatment (Su) used 20μm double-nylon mesh on one side of the baffle plate between the competition compartment and one planting compartment and a 0.45μm nylon mesh on the other side between the competition compartment and the other planting compartment, allowing the AM mycelium of one planting compartment of *E*. *adenophorum* or *A*. *annua* to pass through the competition compartment to utilize nutrients. (3) the non-utilization of nutrients treatment (Nu) which in contrast to Cc and Su, used the 0.45μm nylon mesh to separate both planting compartments from the central compartment, so that the nutrient resources of the competition compartment could not be utilized by both species as their associated mycelia could not cross the fine 0.45μm nylon mesh.

The plant growing substrate was a mixture of limestone soil and sand by volume ratio of 3:1, which had pH 7.45, total nitrogen 2.27 g. kg^-1^, available nitrogen 127.48 mg. kg^-1^, total phosphorus 0.90 g. kg^-1^, available phosphorus 11.48 mg. kg^-1^, total potassium 4.99 g. kg^-1^ and available potassium 287.30 mg. kg^-1^, following the method of measurement of Tan (2005) [36]. The substrate was sterilized with a pressure of 0.14 Mpa and a temperature of 124°C-126°C for one hour before the beginning of the experiment, and the limestone soil was collected from a typical karst habitat near Guiyang city. Seeds of *E*. *adenophorum* and *A*. *annua* were collected from Guanling county of Guizhou province of China, a typical desertification area within a karst ecosystem. In our previous field survey, the *E*. *adenophorum* has severely invaded the karst area of southwest China and coexists with native *A*. *annua*, and both species are herbaceous plants of *Asteraceae* family and have a similar niche [37]. The seeds were sterilized with a 10% hydrogen peroxide (H_2_O_2_) solution for 10 minutes and repeatedly washed with sterile water three times. Each compartment was filled with 2.5 kg of the sterilized substrate, and five seeds of plants were taken into planting compartments, respectively. 50g of inoculum was added into the planting compartments as M^+^ treatments. The inoculum had been propagated with *Trifolium repens* for four months, which was sterilized at 0.14 Mpa, at 126 °C for one hour before inoculation with *Glomus etunicatum*. Additionally, M^-^ treatments received an additional 10 ml of the filtrate by weighing 50g of *Glomus etunicatum* inoculum and by filtering it with ultrapure water, and double-layer filter paper, along with a 50g of autoclaved inoculum was added, in order to ensure the same microflora in M^-^ and M^+^ except for the target fungus (*Glomus etunicatum*). After ten days of seedling growth, two seedlings were kept in each planting compartment. Each treatment used six replicates. All experimental materials were cultured in a greenhouse for 12 weeks and then were harvested for measurement.

### Measurements of mycorrhizal colonization rate, dry weight, nitrogen, and phosphorus

The determination of mycorrhizal colonization rate adopted methods described by He and Zhong (2012) [38]. The root dry weight of *E*. *adenophorum* and *A*. *annua* was determined by weighing root material after drying at 80°C for constant weight. Plant N was determined by the Kjeldahl method and P was determined by Molybdenum-Antimony colorimetry [39]. Plant root morphological indices were measured using a root scanning analysis system (STD1600 Epsom USA; WinRhizo Version 410B) to obtain root length, average diameter, surface area, volume, branching points and tips of roots. The specific root length, area and volume were calculated by the root length, area and volume divided by the root dry weight, respectively [40]. The root N and P content per length, area and volume were calculated by the root N and P content divided by the root length, area and volume, respectively.

### Statistical analyses

Statistical analyses were performed using the SPSS 18.0 software. All of the data were tested for normality and homogeneity of variance before analysis. Variance analysis was applied to compare differences between M^+^ and M^-^ or treatments of Cu, Su and Nu treatments in length, average diameter, surface area, volume, tips and branching points of roots, specific root length, area and volume, and root N and P contents per length, area and volume by the least significant difference (LSD). Three-way ANOVAs were applied for the effects of species origin (*E*. *adenophorum* (invasive) vs. *A*. *annua* (native)), mycorrhizal fungus (M^+^ vs. M^-^) and nutrient access treatments (Cu vs. Su vs. Nu) and their interactions on the root traits of morphology and nutrients. Origin 8.0 software was used to the bar graphs.

## Results

### The mycorrhizal colonization and root dry weight of invasive *E*. *adenophorum* and native *A*. *annua*

**Fig 2:**
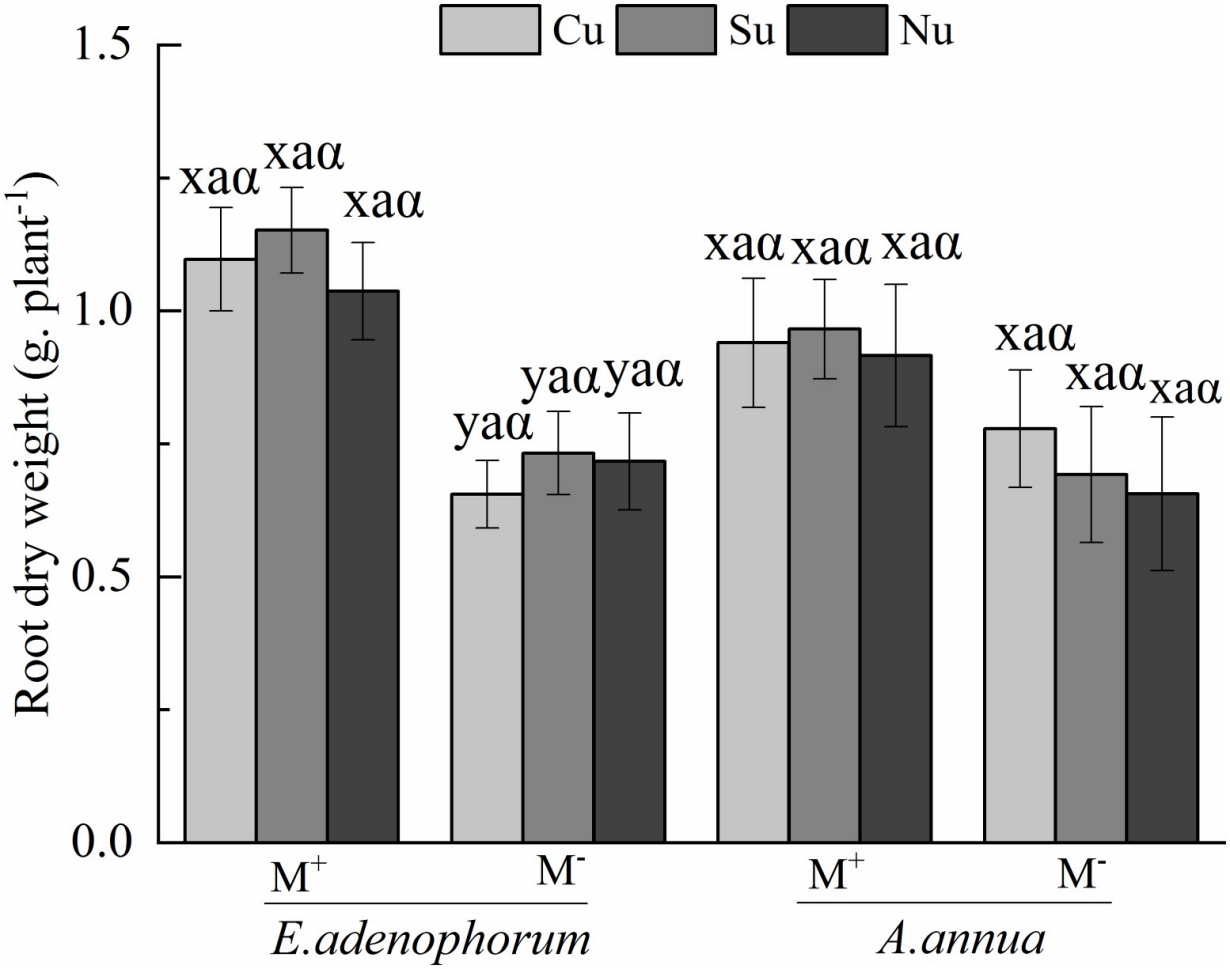
Root dry weight of invasive *E*. *adenophorum* and native *A*. *annua*. Abbreviations: M^+^ = Mycorrhizal fungus was used to inoculate seedlings of *E*. *adenophorum* and *A*. *annua*; M^-^ = Mycorrhizal fungus was not used to inoculate seedlings of *E*. *adenophorum* and *A*. *annua*; Cu = Competitive utilization nutrient treatment; Su = Single utilization nutrient treatment; Nu = Non-utilization nutrient treatment. Lowercase letters (x, y) indicate significant differences between M^+^ and M^-^ treatments of invasive *E*. *adenophorum* and native *A*. *annua* at the 0.05 level; lowercase letters (a, b, c) indicate that there are significant differences among Cu, Su and Nu treatments for invasive *E*. *adenophorum* and native *A*. *annua* at the 0.05 level; Greek alphabet (α, β) indicate that there are significant differences between *E*. *adenophorum* and *A*. *annua* at the 0.05 level.

For the M^+^ treatment, the mycorrhizal colonization rates of *E*. *adenophorum* and *A*. *annua* were not significantly different among Cu, Su and Nu treatments, while no AM colonization was observed under the M^–^. The mycorrhizal colonization rate of *E*. *adenophorum* was significantly greater than *A*. *annua* under the Cu, Su, and Nu treatments, indicating that *E*. *adenophorum* is more responsive to mycorrhizal colonization than *A*. *annua* (Table 1). The species (S) and AM fungus (M) significantly affected the plant's root dry weight (Table 2). For *E*. *adenophorum*, the M^+^ treatment was significantly greater than M^-^ under Cu, Su and Nu conditions; for *A*. *annua*, there was no significant difference between M^+^ and M^-^ (Fig 2). The nutrient access treatments had no significant effects on plants root dry weight (Table 2); there were no significant differences in both species among Cu, Su and Nu treatments under M^+^ and M^-^ (Fig 2). The interaction of S × M significantly affected plants' root dry weight (Table 2). Overall, the root dry weight of *E*. *adenophorum* treated with AM fungus was greater than *A*. *annua*, and this result suggests that AM fungi may have a greater promotion effect on *E*. *adenophorum*.

**Table 1:** The mycorrhizal colonization rate (%) of *E*. *adenophorum* and *A*. *annua*.

Treatments	*E*. *adenophorum*		*A*. *annua*	
	M^+^	M^-^	M^+^	M^-^
Cu	72.1±1.8axα	0	63.0±0.8ayβ	0
Su	72.5±1.7axα	0	65.2±2.0ayβ	0
Nu	71.9±0.5axα	0	62.3±1.6ayβ	0

M^+^ = *E*. *adenophorum* and *A*. *annua* were inoculated with mycorrhizal fungus; M^-^ = *E*. *adenophorum* and *A*. *annua* were not inoculated with a mycorrhizal fungus. Cu = Competitive utilization nutrient treatment; Su = Single utilization nutrient treatment; Nu = Non-utilization nutrient treatment. The values are “mean ± SE”

**Table 2:** Three-way ANOVAs for the effects of species origin (*E*. *adenophorum* vs. *A*. *annua*), mycorrhizal fungus (M^+^ vs. M^-^) and nutrient access (Cu vs. Su vs. Nu) on root dry weight, tips and branching points.

Factors	df	Root dry weight (g. plant^-1^)		Root tips (numbers. Plant^-1^)		Root branching points (numbers. Plant^-1^)	
		*F*	*P*	*F*	*P*	*F*	*P*
S	1	4.812	0.032*	24.219	0.000***	14.21	0.001**
M	1	75.677	0.000***	255.846	0.000***	612.598	0.000***
N	2	0.497	0.611	10.007	0.001**	11.041	0.000***
S × M	1	5.753	0.02*	8.71	0.007*	9.436	0.005**
S × N	2	0.717	0.492	0.769	0.475	1.009	0.379
M × N	2	0.171	0.843	4.327	0.025*	4.821	0.017*
S × M × N	2	1.066	0.351	0.786	0.467	1.854	0.178

Abbreviations: S = Species; M = Mycorrhizal fungus treatments; N = Nutrient access treatments.

* or ** or *** indicates a significant difference at P < 0.05 or P < 0.01 or P < 0.001

### The length, surface area, average diameter and volume on roots of invasive *E*. *adenophorum* and native *A*. *annua*

**Fig 3:**
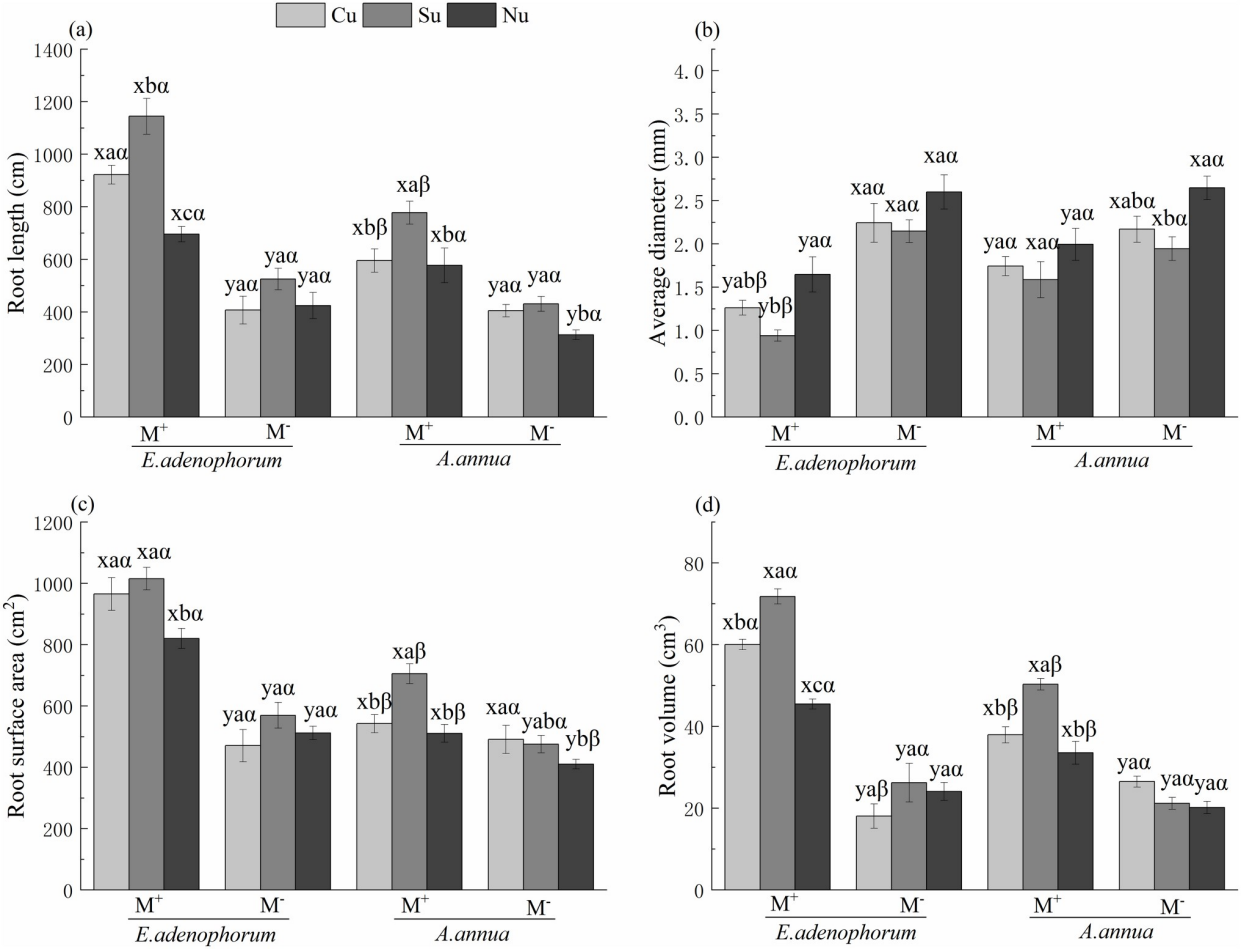
Phenotypic traits of roots of invasive *E*. *adenophorum* and native *A*. *annua*. See Fig 1 for an explanation of M^+^, M^-^, Cu, Su and Nu, lowercase letters (a, b, c) and (x, y) and Greek alphabet (α, β).

The species (S) and AM fungus (M) significantly affected the morphological traits of roots (Table 3). For *E*. *adenophorum*, the length, surface area and volume in roots of the M^+^ treatment were significantly greater than M^-^, but the average diameter of M^+^ treatment was significantly lower than M^-^; for *A*. *annua*, a significant difference was observed between M^+^ and M^-^ in length and volume under Cu, Su and Nu treatments, in surface area under Su and Nu (M^+^ > M^-^), and in average diameter under Cu and Nu (M^+^ < M^-^) (Fig 3a–3d). The nutrient access (N) also significantly affected plant root morphology (Table 3). For *E*. *adenophorum*, under M^+^, the length and volume were significantly different among Cu, Su and Nu treatments (Su > Cu > Nu), the average diameter of the Cu treatment was significantly lower than in Nu, and the surface area of the Su and Cu treatments was significantly higher than in Nu; under M^-^, there were no significant differences in all these root morphological indexes among Cu, Su, and Nu treatments (Fig 3a–3d). For *A*. *annua*, under M^+^, the length, surface area and volume of Su treatment were significantly greater than Cu and Nu; under M^-^, root length in Cu and Su treatments was higher than in Nu, and the surface area of the Cu treatment was greater than in Nu (Fig 3a, 3c, 3d). The interaction of S × M significantly affected morphological traits of roots; and the interactions of M × N and S × M × N significantly affected the root length, surface area and volume (Table 3).

**Table 3:** Three-way ANOVAs for the effects of species origin (*E*. *adenophorum* vs. *A*. *annua*), mycorrhizal fungus (M^+^ vs. M^-^) and nutrient access (Cu vs. Su vs. Nu) on root length, average diameter, surface area and volume.

Factors	df	Root length (cm)		Average diameter (mm)		Root surface area (cm^2^)		Root volume (cm^3^)	
		*F*	*P*	*F*	*P*	*F*	*P*	*F*	*P*
S	1	44.042	0.000***	7.156	0.013*	207.378	0.000***	53.965	0.000***
M	1	206.026	0.000***	96.121	0.000***	371.593	0.000***	454.943	0.000***
N	2	24.458	0.000***	18.31	0.000***	27.759	0.000***	27.766	0.000***
S × M	1	15.403	0.001**	13.345	0.001**	105.247	0.000***	51.883	0.000***
S × N	2	1.705	0.203	0.01	0.991	0.009	0.991	2.431	0.109
M × N	2	5.945	0.008*	0.15	0.861	7.498	0.003**	20.493	0.000***
S × M × N	2	3.678	0.04*	1.038	0.369	7.474	0.003**	6.677	0.005**

Abbreviations: S = Species; M = Mycorrhizal fungus treatments; N = Nutrient access treatments. See Table 2 for an explanation of *, ** and ***

Apparently, under M^+^, the *E*. *adenophorum* was significantly greater than the *A*. *annua* in length under the Cu and Su, and in surface area and volume under the Cu, Su and Nu treatments; but the average diameter of *E*. *adenophorum* was lower than *A*. *annua* under the Cu and Su treatments; under M^-^, the volume of *E*. *adenophorum* was lower than *A*. *annua* under the Cu treatment (Fig 3a–3d). These results indicate that AM fungi can promote the growth and development of the root of *E*. *adenophorum* and *A*. *annua* by changing the root traits and that the root system of *E*. *adenophorum* experienced greater changes in traits reflecting greater enhanced competitiveness than *A*. *annua* when the two species compete for common nutrients in the middle compartment via their mycelium.

### Numbers of root tips and branching points of invasive *E*. *adenophorum* and native *A*. *annua*

**Fig 4:**
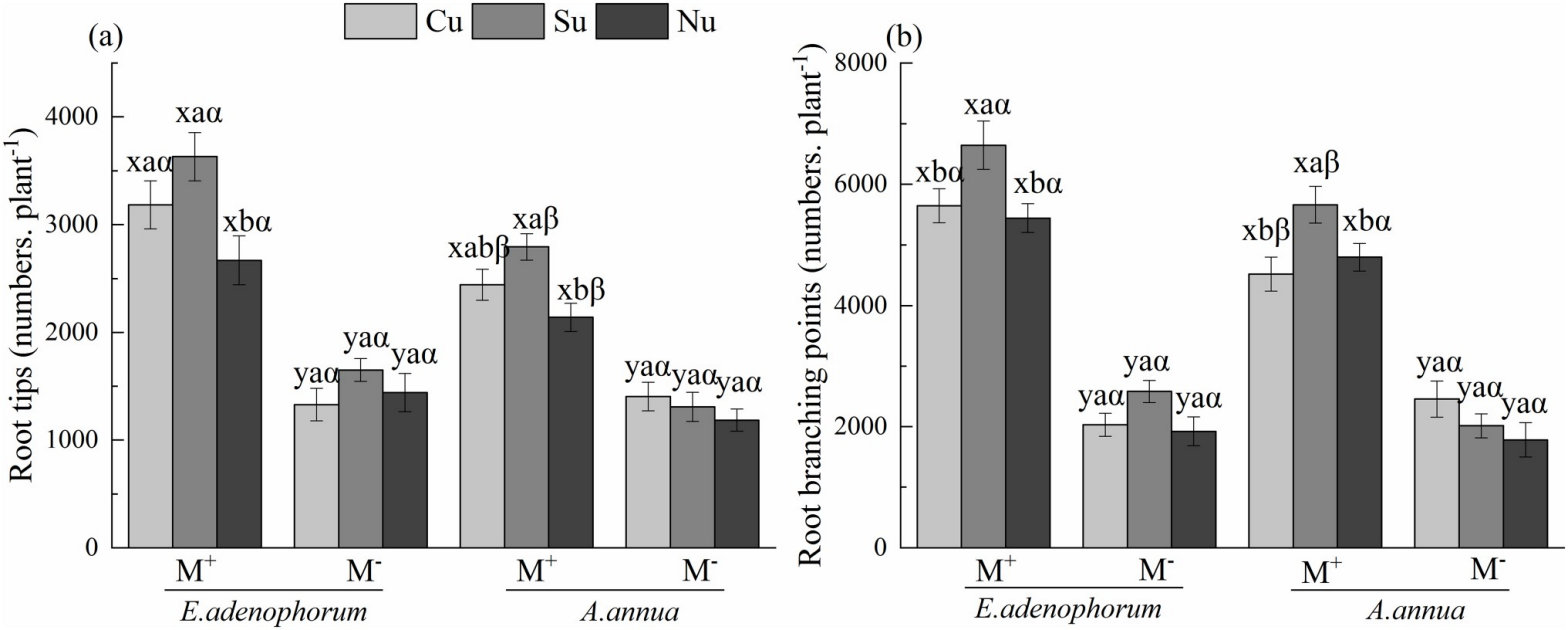
Numbers of root tips and branching points of invasive *E*. *adenophorum* and native *A*. *annua*. See Fig 1 for an explanation of M^+^, M^-^, Cu, Su and Nu, lowercase letters (a, b, c) and (x, y) and Greek alphabet (α, β).

The species (S) and AM fungus (M) significantly affected the numbers of root tips and branching points of plants (Table 2). A significant difference between M^+^ and M^-^ was observed (M^+^ > M^-^) in numbers of root tips and branching points of *E*. *adenophorum* and *A*. *annua* under the Cu, Su and Nu treatments (Fig 4a and 4b). The nutrient access (N) significantly affected the number of root tips and branching points (Table 2). Under M^+^, for *E*. *adenophorum*, the number of root tips in Cu and Su treatments was significantly higher than in Nu, and the number of branching points in Su was significantly higher than in Cu and Nu; for *A*. *annua*, the number of root tips in the Su treatment was significantly greater than in Nu and the number of branching points in the Su was significantly higher than in Cu and Nu; under M^-^, there were no significant differences in root tips and branching points of the two species among Cu, Su and Nu treatments (Fig 4a and 4b). Interestingly, the interactions of S × M and M × N significantly affected the number of root tips and branching points, which indicated invasive plants had a better performance of roots than native plant in M^+^ compared with M^-^ (Table 2). Overall, under M^+^, the number of root tips and branching points of *E*. *adenophorum* were higher than *A*. *annua* under the Cu, Su and Nu treatments; under M^-^, these root indexes of *E*. *adenophorum* was lower than *A*. *annua* under the Cu (Fig 4a and 4b). This result indicates that AM fungi can promote the lateral root growth of both species and that the number of root tips and branching points of *E*. *adenophorum* were more significant than *A*. *annua* when the two species have access to the competition compartment through mycelium.

### Specific root traits of invasive *E*. *adenophorum* and native *A*. *annua*

**Fig 5:**
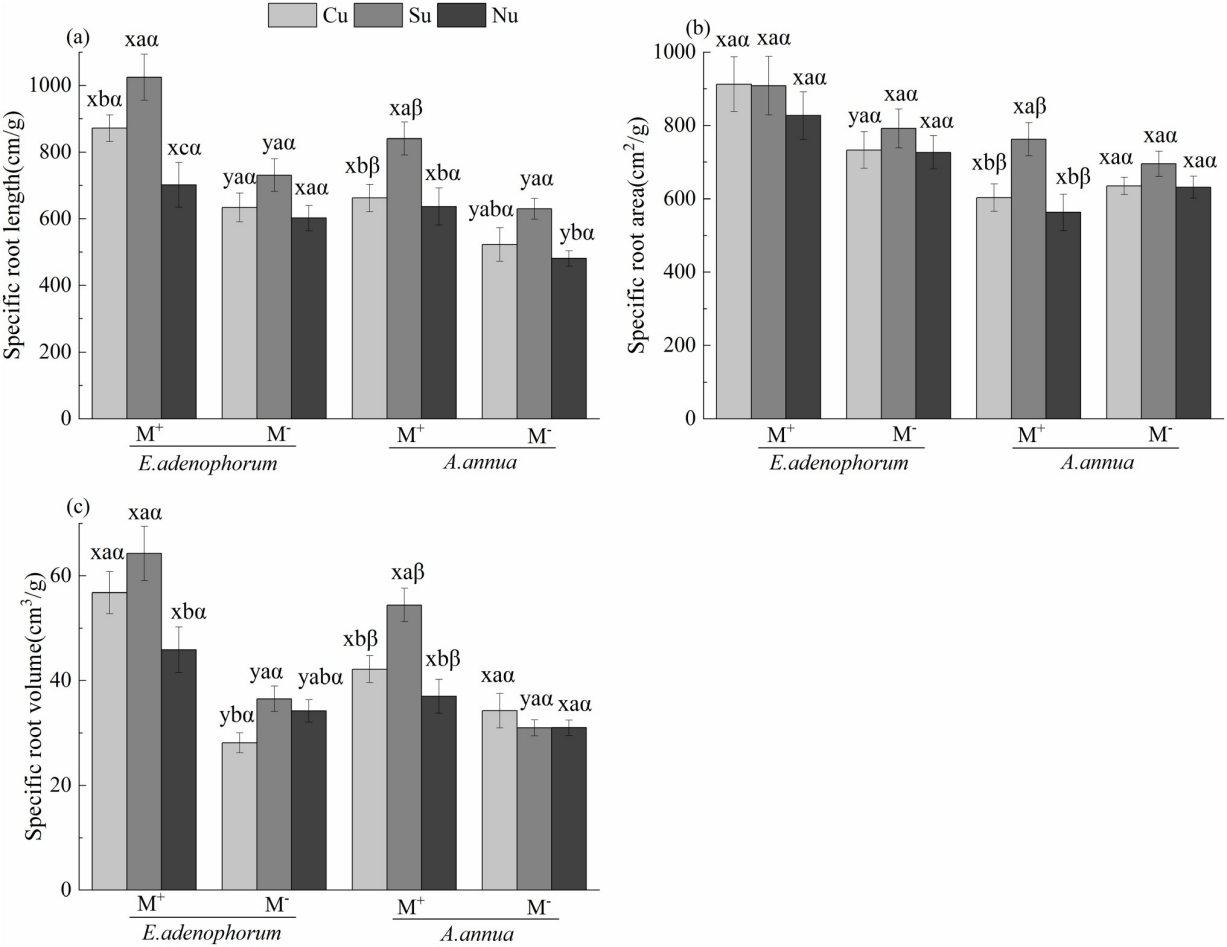
Specific root traits of invasive *E*. *adenophorum* and native *A*. *annua* plants. See Fig 1 for an explanation of M^+^, M^-^, Cu, Su and Nu, lowercase letters (a, b, c) and (x, y) and Greek alphabet (α, β).

The species (S) and AM fungus (M) significantly affected the specific root traits of plants (Table 4). For *E*. *adenophorum*, a significant difference was observed between M^+^ and M^-^ (M^+^ > M^-^) in specific root length under the Cu and Su treatments, in specific root area under the Cu, and in specific root volume under the Cu, Su and Nu treatments; for *A*. *annua*, there was a significant difference between M^+^ and M^-^ (M^+^ > M^-^) in specific root length under the Cu, Su and Nu treatments and in specific root volume under the Su (Fig 5a–5c). The nutrient access (N) significantly affected the specific root traits of plants (Table 4). For *E*. *adenophorum*, there was a significant difference in specific root length among Cu, Su and Nu treatments (Su > Cu > Nu), specific root volume in Cu and Su was significantly greater than in the Nu under M^+^; for *A*. *annua*, all specific root traits of the Su treatment was significantly higher than in Cu and Nu under M^+^ (Fig 5a–5c). The interaction of S × M significantly affected specific root area and volume, which indicated invasive plant had better root traits than native plant in M^+^ compared with M^-^; and the interaction of M × N significantly affected specific root volume (Table 4). Generally, under M^+^, the *E*. *adenophorum* was significantly greater than the *A*. *annua* in specific root length under the Cu and Su treatments, and in specific root area and volume under the Cu, Su and Nu; under M^-^, there was no significant difference in specific root traits between *E*. *adenophorum* and *A*. *annua* under Cu, Su and Nu treatments (Fig 5a–5c). These results indicate that AM fungi can change the specific root traits of *E*. *adenophorum* and *A*. *annua*, the promotion of these traits in *E*. *adenophorum* was more obvious than in *A*. *annua* when both species competed for nutrients in the middle compartment through mycelium.

**Table 4:** Three-way ANOVAs for the effects of species origin (*E*. *adenophorum* vs. *A*. *annua*), mycorrhizal fungus (M^+^ vs. M^-^) and nutrient access (Cu vs. Su vs. Nu) on specific root traits.

Factors	df	Specific root length (cm/g)		Specific root area (cm^2^/g)		Specific root volume (cm^3^/g)	
		*F*	*P*	*F*	*P*	*F*	*P*
S	1	23.73	0.000***	31.864	0.000***	11.603	0.001**
M	1	48.886	0.000***	4.125	0.047*	99.855	0.000***
N	2	19.262	0.000***	4.145	0.021*	10.175	0.000***
S × M	1	0.6	0.442	5.763	0.02*	8.52	0.005**
S × N	2	0.532	0.59	0.693	0.504	0.333	0.718
M × N	2	1.766	0.18	0.579	0.564	7.583	0.001**
S × M × N	2	0.805	0.452	0.679	0.511	2.248	0.115

Abbreviations: S = Species; M = Mycorrhizal fungus treatments; N = Nutrient access treatments. See Table 2 for an explanation of *, ** and ***

### N and P contents in roots of invasive *E*. *denophorum* and native *A*. *annua*

**Fig 6:**
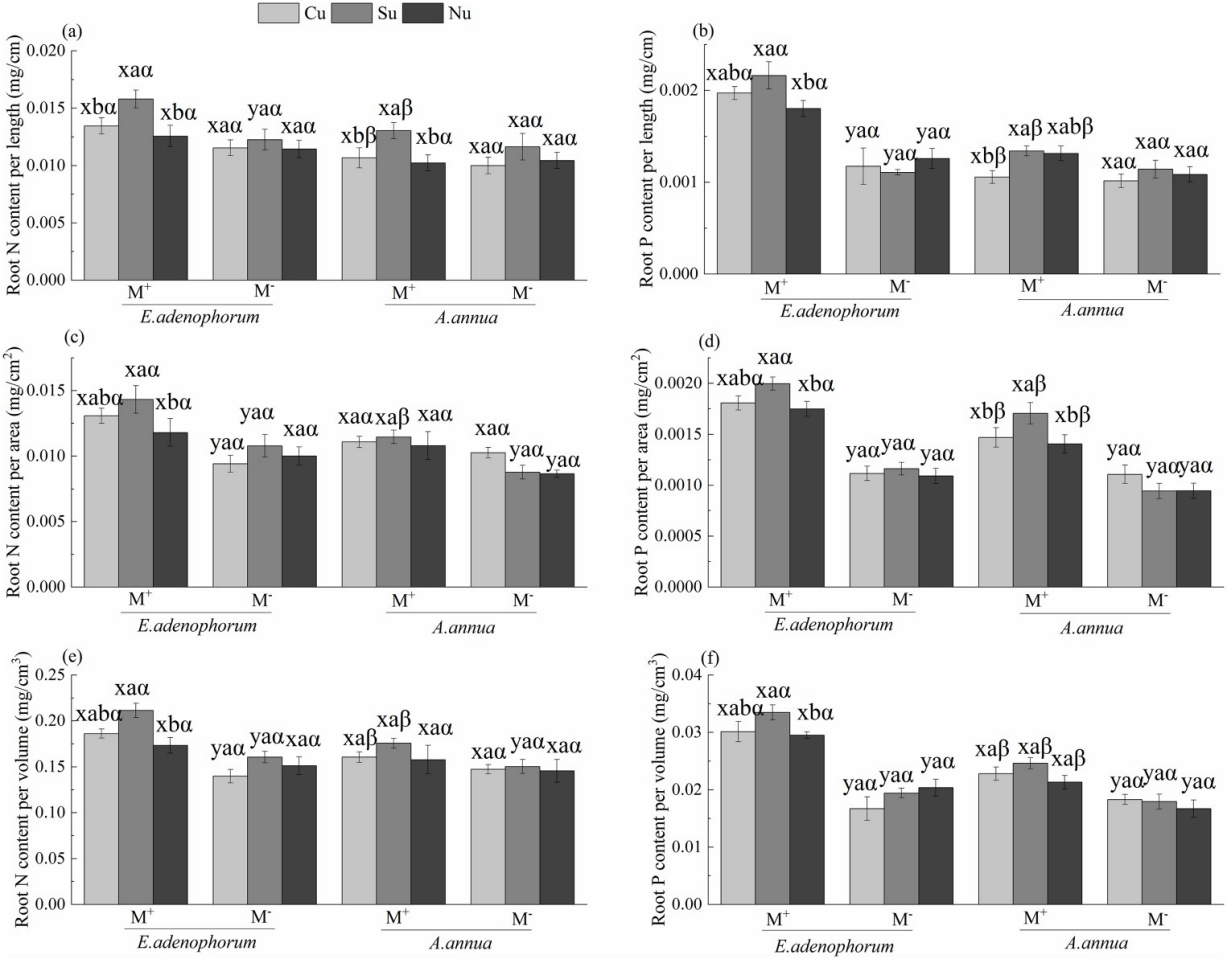
N and P contents in roots of invasive *E*. *adenophorum* and native *A*. *annua*. See Fig 1 for an explanation of M^+^, M^-^, Cu, Su and Nu, lowercase letters (a, b, c) and (x, y) and Greek alphabet (α, β).

The species (S) and AM fungus (M) significantly affected N and P contents in roots of plants (Table 5). For *E*. *adenophorum*, a significant difference was observed between M^+^ and M^-^ (M^+^ > M^-^) in root N content per length under the Su treatment, in root N content per area and volume under the Cu and Su, and in root P content per length, area and volume under the Cu, Su and Nu treatments (Fig 6a–6f). For *A*. *annua*, there was a significant difference between M^+^ and M^-^ (M^+^ > M^-^) in root N content per area under the Su and Nu treatments, in root N content per volume under the Su treatment, and in root P per area and volume under the Cu, Su and Nu treatments (Fig 6c–6f). The nutrient access (N) significantly affected root N per length and volume and root P per area of plants (Table 5). Under M^+^, for *E*. *adenophorum*, root N per length of the Su treatment was significantly greater than the Cu and Nu, the root N per area and volume and root P per length, area and volume of Su treatment were significantly higher than in Nu; for *A*. *annua*, the root N per length and P per area of the Su treatment were significantly greater than Cu and Nu, and the root P per length of the Cu treatment was significantly lower than the Su (Fig 6a–6f). Besides, the interactions of S × M and M × N significantly affected plant root P content, which indicated invasive plants had higher P utilization than native plants in M^+^ compared with M^-^ (Table 5). Generally, under the Cu, under M^+^, N and P contents in roots of *E*. *adenophorum* was higher than *A*. *annua*; under M^-^, there was no significant difference between the two species in root N and P (Fig 6a–6f). It indicates that AM fungi differentially increased the N and P contents in the roots of both species. The absorption capacity of N and P nutrients of the roots of *E*. *adenophorum* was stronger than *A*. *annua* when the two species competed for resources in the middle compartment through the mycorrhizal network.

**Table 5:** Three-way ANOVAs for the effects of species origin (*E*. *adenophorum* vs. *A*. *annua*), mycorrhizal fungus (M^+^ vs. M^-^) and nutrient access (Cu vs. Su vs. Nu) on root N and P contents.

Factors	df	Root N content per length (mg/cm)		Root P content per length (mg/cm)		Root N content per area (mg/cm^2^)		Root P content per area (mg/cm^2^)		Root N content per volme (mg/cm^3^)		Root P content per volme (mg/cm^3^)	
		*F*	*P*	*F*	*P*	*F*	*P*	*F*	*P*	*F*	*P*	*F*	*P*
S	1	15.418	0.001**	56.571	0.000***	11.15	0.003**	23.851	0.000***	6.194	0.02*	37.854	0.000***
M	1	8.957	0.006*	73.052	0.000***	34.111	0.000***	186.89	0.000***	24.691	0.000***	133.687	0.000***
N	2	7.282	0.003**	1.919	0.169	2.025	0.154	3.753	0.038*	3.884	0.035*	2.736	0.085
S × M	1	2.789	0.108	33.111	0.000***	1.789	0.194	4.741	0.04*	4.016	0.056	21.042	0.000***
S × N	2	0.123	0.884	1.209	0.316	1.713	0.202	0.315	0.733	0.599	0.557	1.5	0.243
M × N	2	1.544	0.234	1.803	0.186	0.676	0.518	3.491	0.047*	1.126	0.341	1.791	0.188
S×M× N	2	0.092	0.913	2.21	0.132	1.254	0.303	0.651	0.531	0.359	0.702	0.729	0.493

Abbreviations: S = Species; M = Mycorrhizal fungus treatments; N = Nutrient access treatments. See Table 2 for an explanation of *, ** and ***

## Discussion

AM fungi differentially increased the dry weight, length, surface area, volume, tips, branching points and N and P contents in roots for invasive *E*. *adenophorum* and native *A*. *annua* in this experiment (Figs 2, 3a, 3c, 3d, 4a, 4b and 6a–6f). Previous studies demonstrated that AM mycelia can complement plant roots to expand the absorption range from soil to improve plant nutrient [41]. For instance, AM fungi could obtain N from organic matter and transfer it to host plants [42], and could enhance P uptake for the invasive plant *Microstegium vimineum* [43]; Huang et al. (2011) [44] also discovered that AM fungi facilitated uptake by *A*. *annua* roots for soil N and P nutrients. These studies indicate that AM fungi play important roles in regulating nutrients of N and P for host plants, including invasive or native species, and our experimental results also verified this by AM fungi enhancing the N and P contents of roots in both species. Besides, AM fungi can promote the root growth of *E*. *adenophorum* and *A*. *annua* according to results from Figs 2–4 in this experiment, consisting of the root morphology changes and root biomass enhancement of seedlings that promote growth and development via AM fungi as similarly documented by Lü and Wu (2017) [45] and Liu et al. (2016) [46]. Root morphological plasticity, when associated with AM fungi, may be more substantial in karst habitats with limited nutrients. For example, Yang et al. (2017) [47] discovered that inoculation with AM fungi markedly increased the root length, surface area and volume of *Cinnamomum camphora* seedlings in karst soil; Zhang et al. (2015) [48] showed that AM fungi significantly enhanced the total root length, surface area and volume of *Cyclobalanopsis glauca* in karst rocky desertification areas. Root morphology can reveal a plant’s ability to absorb nutrients [49], and different plant species vary in the plasticity of their root morphology when in association with microorganisms or in response to other factors [50, 51]. Our results indicated that invasive plants overall exhibited better performance of root traits and nutrients than co-occurring common native plant in karst region. This is consistent with findings of previous studies comparing invasive and native species [52–54]. Interestingly, we found that root traits and nutrient utilization of invasive plant were greater than native plant in M^+^ compared with M^-^. This is similar to Li et al. (2016) [55] suggest that mycorrhizal colonization promoted invasive plants to have greater nutrients uptake and competitiveness than native plants, and Zhang et al. (2018) [56] also documented that AM fungi rendered invasive species presenting superior plant traits compared with native species. Together, these results indicated that invasive plant is competitively superior over the co-occurring native plant when with AM fungi.

He and Zhong (2012) [38] revealed that root average diameter and number of tips are parameters reflecting root absorption efficiency. However, Fitter et al. (1994) [57] argued that fine roots have low input, large surface area and short life, while thicker roots grow fast and have a long life, but has a relatively small surface area, so fine roots have more robust uptake capacity. In this study, AM fungi decreased the root average diameter, and significantly increased the number of root tips of *E*. *adenophorum* and *A*. *annua* seedlings (Figs 3b and 4a), which indicated that AM fungi can enhance the root absorption area and efficiency of invasive and native plants in nutrient-deficient karst soil. The greater the specific root length and area, the greater the ability of fine roots to absorb nutrients and water [58]. Wang et al. (2016) [59] confirmed that AM fungi had a significant effect on the specific root length and area of *Sinocalycanthus chinensis*. The AM fungus in this study differentially improved the specific root length of invasive *E*. *adenophorum* and native *A*. *annua*, and enhanced the specific root area of *E*. *adenophorum* (Fig 5a and 5b) and further this study showed that these increases were greater in invasive *E*. *adenophorum* as compared to native *A*. *annua*. Research suggested that plants are more likely to increase mycorrhizal dependence under nutrient deficient conditions, but decrease mycorrhizal dependence under sufficient nutrient conditions [21], which indicating that the invasive *E*. *adenophorum* and the native *A*. *annua* respectively increased their root dry weight depending on AM fungus compared M^+^ with M^-^, as well as *E*. *adenophorum* presenting greater mycorrhizal dependence than *A*. *annua* in limited nutrients karst soil (Fig 2).

AM fungi affected plant competition on nutrient uptake [60] and enhanced the invasiveness of alien plants competing with native plants [13], which probably is mediated by mycorrhizal networks among plant species [61]. Our study found that root dry weight and root N and P of invasive *E*. *adenophorum* and native *A*. *annua* in the Su treatment were greater than the Nu treatment (Figs 2 and 6), which indicates that the epitaxial mycelium of roots can obtain the resources of the competitive compartment outside of the root system to promote the biomass and nutrient accumulation of host plants. AM fungi regulate competition among host plants by reallocating soil resources through mycorrhizal networks [62]. Weremijewicz et al. (2016) [12] found that common mycorrhizal networks can amplify competition by preferential mineral nutrient allocation to large host plants, and Awaydul et al. (2019) [14] showed that common mycorrhizal networks preferentially transferred mineral nutrients to the invasive species, but inhibited the nutrient uptake of native species. These also explain that the mycorrhizal networks in our study tend to allocate more biomass and nutrients to *E*. *adenophorum* in order to obtaining a greater competitive advantage for that species than *A*. *annua* under the Cu treatment in karst soil (Figs 2 and 6). The root dry weight and root N and P of both species in the Cu treatment were lower than in Su, which may be due to competitive inhibition caused by different plant species competing for shared resources [63].

Additionally, AM fungi will inevitably cause changes in plant phenotype while improving plant nutrients [64]. In this study, the length, surface area and volume in roots, the number of root tips and branching points, and specific root length, area and volume of *E*. *adenophorum* and *A*. *annua* in Su treatment were larger than in the Nu treatment (Figs 3–5). These results were similar to Yang et al. (2017) [47] suggest that the root epitaxial mycelia absorbed more nutrients to promote the growth and development of *Cinnamomum camphora* root phenotypes in karst areas; and these root phenotypic indices of *E*. *adenophorum* and *A*. *annua* from Figs 3–5 were that the Cu treatment was lower than Su, which may be caused by both species competing for limited soil resources through the interconnected mycorrhizal network. Plants will maximize resources use to adapt to competition by regulating productivity and root morphology [65]. Research showed that increasing the number of root tips can enhance the ability of plants to use soil resources in situ [66], and the growth and extension of lateral roots can increase the root length and expand the spatial area where plants can utilize soil resources [67, 68]. Root surface area and root length can be used to represent the root competitiveness [22, 69]. In our experiment, the root length, surface area, number of tips and branching points in *E*. *adenophorum* were significantly higher than *A*. *annua* under the Cu treatment (Figs 3a, 3c, 4a and 4b), which indicates that *E*. *adenophorum* may have greater root morphological plasticity response over *A*. *annua* to enhance its competitiveness in order to absorb and utilize the soil resources in karst habitats. Root diameter size determines the utilization efficiency of plant roots for belowground resources, and the uptake capacity of nutrients and water by the smaller diameter roots is higher than the thicker diameter roots [70]. Meanwhile, the smaller the root diameter, the larger the specific root length, indicating that the plant root system has greater uptake ability [49]. In our study, the root average diameter of *E*. *adenophorum* was significantly lower than *A*. *annua*, and the specific root length and area was significantly greater than *A*. *annua* under the Cu condition (Figs 3b, 5a and 5b), suggesting that mycorrhizal networks can confer a greater competitive advantage for the invasive *E*. *adenophorum* in nutrient and water acquisition over native *A*. *annua* root in the fragile karst ecosystems of southwest China.

## Conclusion

In this experiment, AM fungus was associated with changes in root traits and increased nutrient uptake for invasive and native plant species via the interconnective mycorrhizal networks. Specifically, a large number of root traits were enhanced including root dry weight, length, surface area, volume, number of tips and branching points, specific root length and volume, root N and P contents for invasive *E*. *adenophorum* and native *A*. *annua*; and the root average diameter of both species was decreased. Many of the observed increases were more significant for *E*. *adenophorum* than for *A*. *annua*. In conclusion, based on these findings, we suggest that the invasive plant experienced greater benefits than the native plant in nutrient acquisition and for root traits and root system developments when in association with an AM mycorrhizal network in karst habitats.
